# Data on the hemostasis in epistaxis with Topically Administered TXA Versus Topical Oxymetazoline Spray

**DOI:** 10.1016/j.dib.2020.105283

**Published:** 2020-02-13

**Authors:** Kristen Whitworth, Jacob Johnson, Samuel Wisniewski, Meghan Schrader

**Affiliations:** a Lakeland Health, United States; b Michigan State University College of Osteopathic Medicine, United States

**Keywords:** TXA, Tranexamic acid, Epistaxis, Oxymetazoline

## Abstract

The use of tranexamic acid (TXA) has recently gained popularity as a treatment modality for epistaxis in the emergency department. Data are presented on the efficacy of the topical use of the intravenous formulation of TXA versus the vasoconstrictor oxymetazoline applied topically in achieving hemostasis in patient presenting to the emergency department with anterior epistaxis. The original article “Comparative Effectiveness of Topically Administered TXA Versus Topical Oxymetazoline Spray for Achieving Hemostasis in Epistaxis” [1] provides complete interpretation of the data. The dataset regarding these treatment modalities has clinical significance toward preventing an avoidable need for escalation of treatment that could potentially increase patient discomfort and prolong emergency department throughput time.

**Table:** Specifications Table

Subject	Emergency Medicine
Specific subject area	Epistaxis
Type of data	Table Graph
How data were acquired	A standardized form was completed by treating physician. For details, see the attached ‘Data Collection Form’ in supplementary material.
Data format	Raw and analysed
Parameters for data collection	Patients presenting to the emergency department with acute anterior epistaxis that met all inclusion criteria with written informed consent were included. Patients presenting on odd numbered days of the month were assigned to the oxymetazoline treatment group while patients presenting on even numbered days of the month were assigned to the TXA group.
Description of data collection	Patients were assessed for hemostasis after treatment administration at 10, 15, 20, 25 and 30 minutes. If hemostasis was not achieved at 30 minutes, the protocol was aborted, and the second line treatment was chosen by the treating physician. Patients were contacted at 48 hours after discharge for any recurrence of bleeding.
Data source location	Lakeland Health 1234 Napier Avenue Saint Joseph, MI, USA
Data accessibility	Data incorporated within the article
Related research article	Kristen Whitworth, Comparative Effectiveness of Topically Administered TXA Versus Topical Oxymetazoline Spray for Achieving Hemostasis in Epistaxis, Journal of Emergency Medicine, Article in Press

**Table:** 

**Value of the Data** • This dataset compares the effectiveness of the topical application of the intravenous formulation of TXA to the standard of care, oxymetazoline, in achieving hemostasis in acute epistaxis. • The data can be useful for physicians treating patients with acute epistaxis to avoid escalation of treatment to more invasive modalities which can increase discomfort, risk for complications, and time in the emergency department. • This dataset could supplement future investigations on the effectiveness of using TXA in the treatment of acute epistaxis.

## 1 Data description

This dataset was collected from a standardized form (see ‘Data Collection Form’ included in supplemental material) completed by the treating physician for each patient included in this investigation. The raw data regarding each patient is displayed in Table 1 including the age and gender of each patient, prescription for antiplatelet or anticoagulant medications, time to hemostasis, occurrence of rebleeding in the emergency department or at 48 hours, and the second line treatment chosen if hemostasis was not achieved. Tables displaying the analysis of patient characteristics, primary outcomes, secondary outcomes, and second line treatments can be found in the original research article [1].

**Table 1:** Raw data collected from standardized forms completed by the treating physician for both treatment groups.

Treatment Group	Age (Years)	Sex	Anticoagulant or Antiplatelet Therapy	Hemostasis Achieved	Time to Hemostasis (minutes)	Rebleed in the ED	Room to Dispo (minutes)	Rebleed at 48 Hours	Treatment for Failure or Return Visit
TXA	43	F	none	yes	10	yes	151	n/a	4% cocaine
	50	F	none	yes	10	no	92	no	
	79	F	warfarin	no	n/a	n/a	119	n/a	Nasal Packing
	73	M	aspirin	yes	10	no	55	no	
	46	M	none	yes	10	no	109	no	
	75	M	aspirin	yes	15	no	121	no	
	72	M	none	yes	45	no	87	no	
	74	M	warfarin	yes	15	no	104	no	
	84	M	aspirin	yes	35	no	98	no	
	77	m	none	yes	15	no	86	no	
	62	M	none	yes	15	no	108	no	
	85	F	none	no	n/a	n/a	126	n/a	Nasal Packing
	101	F	clopidogrel, aspirin	no	n/a	n/a	140	n/a	Oxymetazoline
	70	F	none	yes	10	no	55	no	
	70	M	apixaban, aspirin	yes	15	no	119	no	
	62	F	none	yes	25	yes	90	n/a	Nasal Tampon
	84	F	warfarin, aspirin	yes	20	no	160	yes	Nasal Tampon
	78	F	clopidogrel	no	n/a	n/a	64	n/a	Nasal Tampon
Oxymetazoline	88	F	apixaban	no	n/a	n/a	129	n/a	Nasal Tampon
	63	F	clopidogrel	no	n/a	n/a	178	n/a	Nasal Tampon
	73	M	rivaroxaban	yes	15	no	106	yes	Nasal Tampon
	50	M	none	no	n/a	n/a	136	n/a	Nasal Tampon
	51	F	warfarin	no	n/a	n/a	162	n/a	Nasal Tampon
	63	F	none	yes	15	no	82	yes	Nasal Tampon
	84	F	warfarin	no	n/a	n/a	76	n/a	Silver Nitrate
	69	M	aspirin	yes	10	no	79	no	
	70	M	prasugrel	no	n/a	n/a	131	n/a	Nasal Tampon, Admission, ENT Consult
	71	M	rivaroxaban	no	n/a	n/a	172	n/a	Nasal Tampon
	62	F	aspirin	no	n/a	n/a	116	n/a	Nasal Tampon
	65	M	dabigatran	no	n/a	n/a	267	n/a	Nasal Tampon
	27	M	none	yes	30	no	115	no	
	86	M	warfarin	no	n/a	n/a	145	n/a	Nasal Packing
	71	F	aspirin	no	n/a	n/a	77	n/a	Pressure
	77	F	apixaban, aspirin	no	n/a	n/a	162	n/a	Nasal Tampon
	74	F	aspirin	no	n/a	n/a	237	n/a	Nasal Tampon
	50	F	clopidogrel	yes	15	no	105	no	
	75	M	warfarin	yes	15	no	119	no	
	48	F	clopidogrel, aspirin	yes	15	no	148	no	

## 2 Experimental design, materials, and methods

This was a prospective collection of data on patients presenting to the emergency department with the chief complaint of epistaxis between January 2016 and January 2018. Patients were enrolled in two community emergency departments, both associated with an emergency medicine residency program. Patients over the age of 18 years were included if they presented to the emergency department with new or acute anterior epistaxis. Exclusion criteria included patients who were pregnant or breast feeding, non-postmenopausal women, patients with surgery to the nose or pharynx within the past three months, concomitant facial injuries from trauma, a history of hemophilia, a blood pressure over 200/120 mmHg or a known allergy to TXA or oxymetazoline. Patients with posterior epistaxis were also excluded from data collection. The determination of posterior epistaxis was made if the patient had blood in the posterior oropharynx on examination after the nasal passages were cleared of blood clots. Patients prescribed oral anticoagulants were not excluded, as a large majority of the patient population were taking these medications. However, the form of anticoagulation (i.e.: aspirin, platelet inhibitors, warfarin, novel factor Xa inhibitors) were noted in patients' charts and documented on the standardized form.

Eligible patients were allocated into two separate treatment groups: topical oxymetazoline or topical application of the intravenous formulation of TXA. Patients presenting on odd numbered days of the month were assigned to the oxymetazoline treatment group while patients presenting on even numbered days of the month were assigned to the TXA group. Prior to application of the assigned drug, the patient was instructed to clear nasal passages of blood clots by blowing his or her nose. Next, suction was applied to affected nare to clear any clots not cleared initially. The treatment drug was then applied to affected nasal passage via aerosolization. The TXA group received 3 mL of intravenous TXA 1000 mg/10 mL through an atomizer in the affected nare. The oxymetazoline group received three sprays of oxymetazoline hydrochloride 0.05% in the affected nare from a standardized 30mL bottle. After application of the topical drug, direct pressure was applied over the cartilaginous portion of the nasal bridge with patient's fingers. The patient was instructed to pinch the area firmly after the proper amount of pressure was demonstrated by the treating physician. Neither the patient nor the physician was blinded to the treatment group due to the differences in the modes of administration of the medications, as TXA was administered through an automizer and oxymetazoline through aerosolized sprays from a standard bottle. The physician assessing the outcome of the treatment was the same individual that administered the treatment drug and therefore was not blinded either. This physician was responsible for completing the standardized form.

Assessment for ongoing bleeding was performed at 10, 15, 20, 25 and 30 minutes after drug administration and application of pressure on the nose. At 30 minutes, if hemostasis had not been achieved, the protocol was aborted, and the physician was left to treat at their own discretion. Patients were contacted by phone by the principal investigator 48 hours after discharge to document any rebleeding.

## Conflict of Interest

The authors declare that they have no known competing financial interests or personal relationships that could have appeared to influence the work reported in this paper.
