# Supplementary material for: Data on the hemostasis in epistaxis with Topically Administered TXA Versus Topical Oxymetazoline Spray
Source: Data Brief. 2020 Feb 13;29:105283. doi: 10.1016/j.dib.2020.105283 (PMC7047014; doi:10.1016/j.dib.2020.105283)
Supplement: Multimedia component 1 [file mmc1.docx]

**TXA in Epistaxis**

Exclusion Criteria: < 18 years of age, posterior bleed, known pregnancy, breastfeeding, childbearing potential, surgery to the nose/pharynx in past 3 months, multiple concomitant facial injuries, history of hemophilia, allergy to TXA or oxymetazoline, BP >200/120

**Odd Days of the Month**: oxymetazoline (Afrin) - 3 puffs

**Even Days of the Month**: IV TXA - 3mL via Intranasal Atomization Device

Protocol:

- Patient signs research consent form
- Instruct patient to blow nose/clots out
- Administer medication and chart time
- Instruct patient to pinch nose with both fingers
- Check for hemostasis at 10, 15, 20, 25 and 30 minutes
- If no resolution at 30 minutes consider failure and treat according to your discretion

Patient Sticker:

Prescribed Oral Anticoagulation or Antiplatelet Medications: Yes or No If yes, type:

Time of Initial Treatment:

Time Hemostasis Achieved:

Rebleed at bleed prior to discharge? Yes or No
